# Sociodemographic Factors Associated with the Knowledge and Use of Birth Control Methods in Adolescents before and after Pregnancy

**DOI:** 10.3390/ijerph16061022

**Published:** 2019-03-20

**Authors:** Reyna Sámano, Hugo Martínez-Rojano, Gabriela Chico-Barba, Bernarda Sánchez-Jiménez, Selene Sam-Soto, Ana Lilia Rodríguez-Ventura, Laura Mejía-Luna, Sylvia Sclavo-Melo

**Affiliations:** 1 Departamento de Nutrición y Bioprogramación, Instituto Nacional de Perinatología, Secretaría de Salud, Montes Urales 800, Lomas de Virreyes, Mexico City C. P. 11000, Mexico; ssmr0119@yahoo.com.mx (R.S.); gabyc3@gmail.com (G.C.-B.); emiberna20@yahoo.com.mx (B.S.-J.); rovalilia@hotmail.com (A.L.R.-V.); 2 Sección de Posgrado e Investigación, Escuela Superior de Medicina del Instituto Politécnico Nacional, Plan de San Luis y Díaz Mirón s/n, Casco de Santo Tomas, Mexico City C. P. 11340, Mexico; 3 Coordinación de Medicina Laboral, Instituto de Diagnóstico y Referencia Epidemiológicos (InDRE) “Dr. Manuel Martínez Báez”, Secretaría de Salud, Francisco de P. Miranda 177, Lomas de Plateros, Mexico City C. P. 01480, Mexico; 4 Coordinación de Colposcopía. Instituto Nacional de Perinatología, Secretaría de Salud, Montes Urales 800 Lomas de Virreyes, Mexico City C. P. 11000, Mexico; selenesams@hotmail.com; 5 Coordinación de Nutrición, Universidad del Valle de México-Chapultepec, Avenida Observatorio 400, 16 de Septiembre, Mexico City C. P. 11810, Mexico; pimpodhumo@gmail.com; 6 Departamento de Salud, Universidad Iberoamericana, Prolongación Paseo de la Reforma 880, Lomas de Santa Fe, Mexico City C. P. 01219, Mexico; sylvisclavo@hotmail.com

**Keywords:** birth control methods, adolescents, pregnancy in adolescents, sex education, Mexico

## Abstract

Adolescent pregnancy rates are high worldwide. However, insufficient information exists regarding the frequency of birth control methods used before the first pregnancy and postpartum. In the current study, we analyzed the association of sociodemographic factors with the knowledge of birth control methods and their use before and after pregnancy in a sample of adolescents in Mexico City. A cohort study was conducted on 600 pregnant adolescents in Mexico City, from 2013 to 2017, at a health care institution providing prenatal care. Participants were assessed during the second trimester and four months postpartum. The questionnaire explored the knowledge of birth control methods, their use, and other associated factors. Two logistic regression models were implemented to identify potential variables associated with the lack of birth control method use before and after pregnancy. The mean age of participants was 15.4 + 1 years, of which, 48% and 65.2% used a birth control method before pregnancy and postpartum, respectively. We found that the main factors associated with increased risk of not using any birth control method before pregnancy included being under the age of 15 years, school dropout, having an educational lag, initiation of sexual life before the age of 15, and having a mother who did not inform their child about contraceptives. By contrast, variables associated with a higher risk of not using any contraceptive methods after pregnancy included educational lag, lower level of education, and the fact that the adolescent had not used any birth control prior to the pregnancy.

## 1. Introduction

In 2015, approximately 16 million adolescents between the age of 15 and 19 years were reported pregnant, and about one million females <15 years gave birth—representing ~11% of births worldwide, according to the World Health Organization (WHO). Of these pregnancies, 95% occurred in poor and developing countries, which have large populations with low or middle income [1,2]. Furthermore, in 2015, the median birth rate worldwide was 49 per 1000 adolescents between the ages of 15 and 19 years [2], while the birth rate of individual countries ranged from 1 to 299 births per 1000 adolescents. The highest birth rate was recorded in Sub-Saharan Africa [3].

Although the use of birth control methods has increased in many regions of the world, especially Asia and some Latin American countries, its use remains low overall. Worldwide, a slight increase was observed in the frequency of birth control method use over the last two and a half decades, from 54% in 1990 to 57.4% in 2015. In different parts of the world, the proportion of women aged 15–49 years using some method birth control either increased slightly or stabilized from 2008 to 2015. In Africa and Asia, this proportion increased from 23.6 to 28.5% and from 60.9 to 61.8%, respectively. In Latin America and the Caribbean, the proportion was stable at 66.7% [4].

It is estimated that in developing countries, ~225 million women want to postpone or avoid pregnancy. Nevertheless, these women do not opt for using an effective method of birth control. There are reasons that may explain this phenomenon: Low educational level [5]; lack of variety in birth control methods; limited access to birth control methods, especially for the adolescents younger than 15 years of age, for women from a low socioeconomic status, and for single women; fear of the adverse effects; religious or cultural beliefs regarding contraception; gender barriers; and poor-quality medical services [6,7,8]. A poor level of knowledge about birth control methods could be associated with unplanned pregnancy.

Educational level is one of the factors associated with the use of contraceptives. Yago-Simón et al. [9] demonstrated that, in a group of 583 women aged 13–24 years, a lower level of education is linked to an elevated risk of unwanted pregnancy, further highlighting risk of low use of birth control methods [4]. Furthermore, previous reports have associated pregnancy in adolescents with low educational levels and dropping out of school [10]. In fact, in Latin America, there is a 5-fold greater likelihood of dropping out of school for pregnant adolescents compared to those who did not conceive [10,11].

The information available regarding the use of birth control methods indicates that adolescents have higher rates of failure compared to adults, in addition to lower rates of continuous use and compliance [12]. Young men are less concerned about contraception, and when faced with unwanted pregnancy often do not assume responsibility [13,14].

In Latin America and the Caribbean, the birth rate among adolescents is 18%, and the fertility rate is the second highest in the world (80 births per 1000 15- to 19-year-old adolescents). Although this rate has decreased globally, the decline has been minimal [15].

The age of sexual activity has declined in Latin America and the Caribbean, depending on gender and socioeconomic level [16], even though births from adolescent mothers in Mexico remain high [17]. It has become harder for adolescent mothers to successfully handle daily situations linked to academic and work-related development [18], a situation that exacerbates gender inequality by creating uncertainty for young women, especially low-income adolescent woman [16,19,20].

Adequate sex education is not provided in many Latin America countries and the Caribbean, including Mexico, creating the situation where some adolescents do not know how to avoid pregnancy [21]. These particular individuals may therefore feel extraordinarily ill-at-ease and embarrassed about requesting birth control services. In some cases, birth control methods are expensive, difficult to obtain, or even illegal for adolescents [22]. Even when sexually active adolescents have easy access to birth control methods, they are less prone than adult women to utilize them. On the other hand, adolescent women are sometimes exposed to situations where they cannot refuse entering into unintended or forced intercourse, both of which usually take place without protection [21].

The knowledge and use of birth control methods in Mexico were evaluated by the National Health and Nutrition Survey in 2012 (in Spanish, “Encuesta Nacional de Salud y Nutrición”, or ENSANUT-2012) [23], which showed that only 33.4% of young women aged 15 to 19 years reported using some birth control method during their first sexual intercourse. Among this group, 80.6% said that they used condoms and 6.2% opted for a hormonal treatment (the pill, injectable hormones, or a patch). Surprisingly, the choice of the hormonal treatment was more common among adolescents aged 12 to 15 years compared to teens aged 16 to 19 years (by almost three percentage points, 10.1 versus 7.3%, respectively). According to the ENSANUT survey, of the total number of sexually active adolescents aged 12 to 19, half of them (51.9%) had previously been pregnant, and 10.7% were pregnant at the time they filled out the questionnaire [13,23].

Despite receiving information from the school and mass media regarding the negative consequences of pregnancy, levels of adolescents’ knowledge with regard to their sexuality and reproductive health remain clearly deficient. In addition, a lack in connecting the understanding of adverse outcomes with behaviors and attitudes of adolescents is evident [24].

In their study, Schor et al. [25] demonstrated inadequate knowledge of contraceptive methods among adolescents aged under 14 (48.3%), which the authors attributed to the lack of knowledge at this age when they are not yet sexually active. However, there was a 55% increase in the percentage of knowledge among adolescents aged 15 and 92% among those aged 19, although the quality of knowledge was not evaluated. Few studies have evaluated the level of knowledge with respect to contraceptive methods using a scoring system [26]. It has been shown that, among adolescent women and women of different ages, knowledge regarding contraceptive methods is low or average in approximately 70% of respondents, which could contribute to its use in adult life [27]. However, the use of contraceptive methods has not been directly associated with personal knowledge, and other elements have been suggested to affect their use, including age at first sexual intercourse, access to contraceptive methods, having a stable sexual partner, refusing the partner’s use of contraceptives, the desire to get pregnant, and poor communication between parents and their children regarding sexual problems [28].

A rising trend of sexual activity during adolescence, poor compliance with modern contraceptives, and inadequate use of family planning services, suggest that the likelihood of getting pregnant more than once during adolescence is high [29].

Factors associated with low contraceptive use after pregnancy are not well known, and few studies examine the initiation of postpartum contraception use in adolescent mothers. In addition, our research group previously reported that adolescents had a good level of knowledge about birth control methods; nevertheless, they were not asked about the specific method used or their primary sources of information [30]. In this paper, we aimed to analyze the association between sociodemographic factors, knowledge and use of birth control methods before and after pregnancy in a sample of adolescent women in Mexico City.

## 2. Material and Methods

### 2.1. Study Design and Subjects

A prospective cohort study was conducted with the participation of pregnant adolescents. The study was accomplished with contribution from the Instituto Nacional de Perinatología (National Institute of Perinatology, INPer) and the Escuela Superior de Medicina, Instituto Politécnico Nacional (Superior School of Medicine, National Polytechnic Institute), both located in Mexico City. INPer is a third-level institution that provides medical care to women from Mexico City and nearby states, who have no social security coverage and are from low–medium socioeconomic status, where teenage pregnancy is prevalent.

The participants were pregnant adolescents who received prenatal medical care at INPer, and were followed-up four months postpartum, from January 2013 to December 2017. Sampling was non-probabilistic, based on consecutive cases that met the following inclusion criteria: Adolescents aged 10–19, first and singleton pregnancy, no chronic or mental disease. The sample size was calculated based on a 1000 finite population (pregnant adolescents that met the inclusion criteria), an expected percentage of adolescents who did not use birth control methods for their first sexual intercourse of 67%, an acceptable error of 3%, and a confidence of 95%. The study required a total of 486 participants. However, 15% was determined as the expected percentage of losses to follow-up; as such, a total of 572 participants were required. During the recruitment period, 750 adolescents agreed to be part of the study. Nevertheless, only 600 adolescents concluded the assessments regarding the use of birth control methods before and after their first pregnancy. This number was intended to over-represent all pregnant adolescents that had prenatal medical care at INPer, and to avoid statistical error. Written informed consent was obtained from the adolescents as well as from their parents or guardians.

### 2.2. Assessments

Trained nurses and physicians from INPer used a written questionnaire. The questionnaire’s content was previously validated by three researchers with expertise in the field. Moreover, experts outside INPer were consulted to ensure that the questions were understandable. The instrument’s design was based on the most common responses obtained in a prior qualitative study involving 39 semi-structured interviews with adolescents, about their sexuality, sociodemographic, family structure, knowledge about the use of different birth control methods, and the source of this information. The next step was to validate the instrument in a group of adolescents that represented the study participants, with the purpose of finding out the consistency of the questionnaire. Cronbach’s alpha value of reliability was 0.80.

The questionnaire consisted of three sections: Section one, regarding sociodemographic and clinical data; Section two assessed the knowledge and sources of information regarding sexuality and birth control methods; and Section three included questions regarding the use of birth control methods. The three sections of the questionnaire were applied at baseline assessment during the second trimester of gestational, and Section three was applied only at four months postpartum.

For Section one, the collected sociodemographic data were age, marital status, occupation, level of education, educational lag, age at menarche, age of initiating sexual activity, number of sexual partners, and family structure. Parents’ information regarding age, level of education, occupation, and at what age they had their first child was also obtained.

Section two consisted of 11 multiple-choice questions regarding what the adolescent knew about menarche, reproductive health, teenage pregnancy, and birth control methods (e.g., *Who gave you the information about birth control methods? What type of information did you receive about your sexuality?*). The participants were also asked about the individuals who were the source of that information (mother, physician, teacher, others).

Section three contained questions about birth control methods (e.g., *What kind of birth control method did you use before pregnancy?*) and if the adolescent had decided to use any of them, as well as what was the source of information leading to the decision of their use. Finally, the participants were asked if the chosen method met her expectations, and if it was suitable for her. This section was conducted twice: At baseline, to gather information about the use of birth control methods before pregnancy, and via a telephone call four months postpartum, to estimae their use after pregnancy.

### 2.3. Operational Definitions

The use of birth control methods was assessed in a direct question: The participant was asked if she had used any birth control method before and after pregnancy. Regarding family structure, a nuclear family was defined as two parents and their children (in this case, the adolescent) living in the same home. Familiy structures that were not nuclear were defined as “others”. The participant’s occupation was defined as student, housewife, or worker. Age of initiating sexual activity was categorized into less than or equal to 15 years and older than 15 years. Planned pregnancy was determined when the adolescent had sexual intercourse with the purpose of getting pregnant.

Educational level was defined as “high” or “low” when the adolescent had completed high or elementary/middle school, respectively. Furthermore, an educational lag was determined when the adolescent’s real age was more than two years greater than the typical age in her current school grade.

The socioeconomic level was evaluated in six categories according to the Mexican Association of Market Survey and Public Opinion (Asociación Mexicana de Investigación de Mercados y Opinión Pública) questionnaire [31]. The categories were high, slightly above the average, average standard of living, slightly below the average, low or austere standard of living, and the lowest income or quality of life. As most women attending INPer come from low–medium socioeconomic status, the categories were recoded into two: Low (low or austere standard of living + the lowest income or quality of life) and average or slightly below (average standard of living + slightly below the average).

### 2.4. Ethical Considerations

Each participant and her parents or guardians provided written informed consent. Data confidentiality and anonymity were guaranteed by using file numbers. This research was approved by the Institutional Review Board (number 212250-49481). To compensate for their participation, the adolescents received free nutritional counseling and informational flyers about proper nutrition during pregnancy—recommendations were made by the official Mexican Norm of the Secretariat of Health (Norma Official Mexicana; NOM-043-SSA2-2005).

### 2.5. Statistical Analyses

Measures of central tendency and dispersion were calculated for quantitative variables, whereas frequencies and percentages were obtained for categorical variables. The sample was divided into users and non-users to compare sociodemographic and knowledge variables, using student’s *t*-test, U-Mann Whitney, and Chi-squared tests. Then, two logistic regression models were performed using the enter method to identify variables associated with the use of birth control methods. The first model was performed by using birth control methods before pregnancy as the dependent variable, and the second was done by using birth control methods after pregnancy as the dependent variable. Statistical significance was considered at *p* < 0.050. The data were analyzed in the SPSS statistical program, version 21 for Windows (IBM^®^ Corp, North Castle, NY, USA).

## 3. Results

### 3.1. Sample Characteristics

A total of 600 pregnant adolescents participated in the study, mean age was 15.4 ± 1 years, 48% used a birth control method before pregnancy, and 21% (*n* = 126) had planned their pregnancy.

The socioeconomic level of 76% of the adolescents was slightly below average, with 24% of the families having low or very low income. Those coming from nuclear families were 48%. After becoming pregnant, 56% of the participants dropped out of school, 25% continued their education, and 19% dropped out before becoming pregnant, because they did not like to study or did not have the financial means to go to school.

Regarding the adolescents’ mothers, 56% had not completed middle school, over half were housewives, 3% were professionals, and the rest had informal jobs. In the case of the adolescents’ fathers, the majority were wage workers, such as factory workers, plumbers, chauffeurs, or construction workers.

We found that 233 participants (39%) had an educational lag, and only 145 (24%) continued in school during their pregnancy. The median gestational age at baseline was 28 weeks. The sociodemographic characteristics of adolescent users and non-users and their parents are shown in Table 1. The information was obtained from 592 mothers and 539 fathers, 50% of the mothers and 27% of the fathers had their first child when they were adolescents.

### 3.2. Knowledge about Sexuality and Birth Control Methods

**Figure 1:**
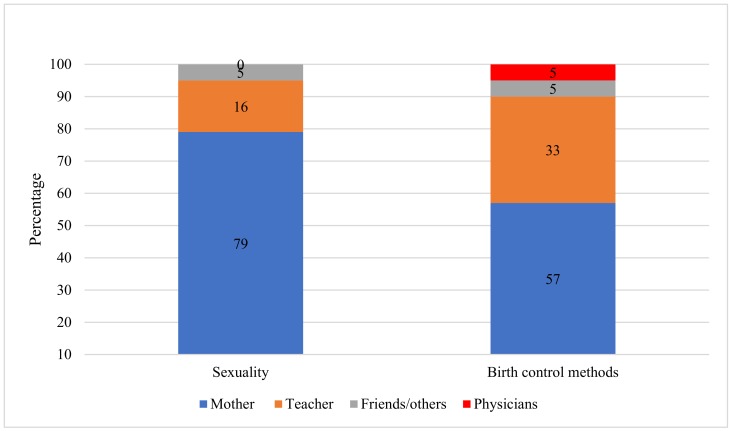
Sources of information about sexuality and birth control methods in a sample of pregnant adolescents.

Of all participants, 94% reported that they had received the necessary information about sexuality at least once, in which case, the mother was the primary source of information for 79% of them, followed by a teacher for 16%, and other informants for the remaining 5%. Regarding the information regarding birth control methods, similar results were observed: Mothers were the primary source of information for 57% of the cases, a teacher for 33%, a physician in 5%, and a friend in 5% of cases (Figure 1).

At the onset of menarche, the adolescents were mainly informed about the importance of self-care and being responsible, the possibility of getting infected with human immunodeficiency virus (HIV), and the use of birth control methods. We found that the frequency of these topics was more common in the group of birth control method users, as shown in Table 2.

Of the 126 (21%) adolescents with planned pregnancies, 56% had received information on sexuality and birth control from their mother. However, the 474 (79%) adolescents who did not plan or want to be pregnant received the information about sexuality and birth control from a teacher, a physician, or a friend (*p* = 0.073).

We found that the use of birth control methods before pregnancy was more frequent in adolescents older than 15 years, with a higher level of education, who received most of their information about sexuality from their mother, and whose mothers were working outside the home. After pregnancy, the statistically significant variables were level of education, educational lag, being a student, and socioeconomic level (Table 3).

Assessment at four months postpartum showed that the adolescents whose mothers had a higher level of education were more likely to use a birth control method (77%) compared to participants whose mothers had a lower level of education (65%, *p* = 0.058).

### 3.3. Use of Birth Control Methods before and after Pregnancy

Before pregnancy, 320 adolescents (53.4%) sometimes used a birth control method, of which the most frequently used were condoms and hormonal methods (the pill, injectable, or a patch), followed by a combination of a hormonal method and condoms. Furthermore, emergency contraception (the morning-after pill) was also used as a birth control method.

**Figure 2:**
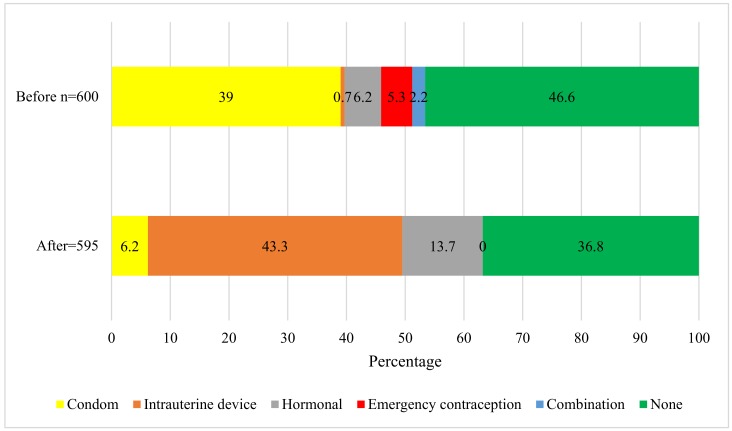
The use of birth control methods before and after pregnancy in a sample of pregnant adolescents.

After pregnancy, only 376 participants (63.2%) used some method of contraception, where the most frequent was the intrauterine device (Figure 2).

Of the adolescents who used a birth control method before pregnancy, 232 (80.6%) decided to continue its use after childbirth. Whereas, of the adolescents who did not use any birth control method before pregnancy, 153 (49%) continued without any after childbirth (*p* = 0.001).

Eighty-five percent of the adolescents using a birth control method postpartum were daughters of women who had a job, versus the 15% who had mothers who were housewives (*p* = 0.020). The variables that increased the odds of not using any birth control method before pregnancy were being under 15 years old (OR: 1.950; CI: 1.284–2.973), having dropped out of school (OR: 2.824; CI: 1.796–4.440), having a low level of education (OR: 1.578; 1.033–2.410), having an educational lag (OR: 2.157; CI: 1.442–3.227), and receiving information from someone other than the mother about birth control methods (OR: 1.054; CI: 1.009–1.102). The variables associated with a higher risk of not using a birth control method postpartum included drop-out of school (OR: 2.823; CI: 1.667–4.780), and that the adolescent had not used any birth control before the pregnancy (OR: 2.392; CI: 1.599–3.578) (Table 4).

## 4. Discussion

The knowledge and frequency of the birth control method used before and after pregnancy were studied in a group of adolescents from Mexico City and other nearby states from 2013 to 2017. The possible associations between the use of birth control methods and other variables were considered. We found that the main factors associated with an increased risk of not using any birth control method before pregnancy were the following: Aged under 15 years, school drop-out, having an educational lag, and having a mother who did not provide information about contraceptives. The variables associated with a higher risk of not using any contraceptive methods after pregnancy were educational lag, lower educational level, and that the adolescent had not used any birth control before the pregnancy.

### 4.1. Sociodemographic and Initiation of Sexual Life

Poor and developing countries are of particular interest when studying adolescent pregnancy, since a considerable proportion of adolescents in those countries initiate sexual life when they are 14 years old or less (whether by their own free will, by force, or by arranged marriage) [3]. In Mexico, ENSANUT-2012 [23] identified a nationwide percentage of 23.5% of adolescents (12–19 years old) had had sexual intercourse at least once. The National Demographic Dynamic Survey of 2014 (Encuesta Nacional de la Dinámica Demográfica 2014, or ENADID-2014) described that the average age of sexual life initiation was 15.9 years for women, half of which initiated their sexual activity before being 15.4 years old [32]. The findings from our study are similar to those described by Rengifo-Reina et al. [33], which was conducted in a group of Colombian adolescents. The age of sexual life initiation in both studies is very similar to the data reported for poor or developing countries.

In Latin America and the Caribbean, between 25 and 38% of 12 to 19 years old are sexually active. Samandari et al. [34], and Gilliam et al. [35] reported that the majority of sexually active adolescents in Mexico, and the rest of Latin America and the Caribbean, used one of the least effective methods of birth control, especially during their first sexual intercourse. This situation shows a desire to avoid unwanted pregnancy, accompanied by insufficient knowledge about the adequate use of different birth control methods. Our study also demonstrates that during their first sexual intercourse, the adolescents chose one of the least effective methods of birth control, such as a condom or a spermicidal ovule, even though there was an apparent desire to avoid pregnancy.

### 4.2. Information about Sexuality and Birth Control Methods

In our sample, we found that >90% of participants reported having had prior knowledge about sexuality and the use of contraceptives. However, research conducted in China showed that half of their sample of adolescents received some information about sexuality, and that the main topic was the use of contraceptives [36]. In Uganda’s adolescents, the percentage was also close to 50% [37].

When comparing adolescents that obtained information about birth control methods from their mother versus from someone else, a statistically significant difference was found regarding the use of contraceptives. Results from our current study are similar to those reported by Rodríguez-Vignoli and Cavenaghi [38], who demonstrated that sex education (including information about different methods of contraception) contributes to a decrease in the number of adolescent pregnancies in Latin America and the Caribbean, especially if the young women receive information from their mothers. Overall, our study shows that almost all participants (94%) obtained information about the different birth control methods.

Regarding sexuality-related information, it was frequent that mothers advised their daughters about the importance of self-care awareness, of being responsible, and the possibility of getting infected with HIV, which was considered as information encouraging the adolescent’s personal hygiene. This type of information is often given by older women, which is similar to the results we obtained [39]. These results show that the primary source of information about sexuality for adolescent girls is the women of their family, in addition to teachers and friends. Therefore, it is reported that adolescents could increase their veridic-scientific knowledge through comprehensive sexual health education in school, which is focused on developing self-esteem and self-efficacy [40,41].

Our results show that self-care was a relevant topic of information for adolescents, which was primarily sourced from their mothers, which is in accordance with data published by Atienzo et al. [42], who demonstrated that educational interventions that include parents favor healthy sexual behavior in adolescents. Therefore, parents’ participation should be included in any intervention concerning adolescents. On the other hand, Swain et al. [43] demonstrated in a group of a thousand parents that their beliefs regarding the use of condoms and the effectiveness, safety, and ease of use of the oral contraceptives are directly related to the amount of sexual communication between parents and adolescents. We believe that educating parents can result in more frequent and accurate sexual communication with adolescents.

In our study, we found that the majority (95%) of participants knew the risk of getting a sexually transmitted infection, such as HIV, which was also reported in Uganda [37] and Romania [44]. This is an important topic, which is contradictory, because in our study more than 90% had prior knowledge that some birth control methods can prevent infections, nonetheless their use was low. Thus, despite their knowledge, <50% used a contraceptive such as condoms. Similar findings were reported in Romanian adolescents [44]. It is essential to consider exploring different factors that can affect the use of birth control methods, since even though adolescents have the knowledge about them, their use remains low, as demonstrated in our current study.

Being responsible was another topic of information that was reported in our study. This responsibility means that the adolescent must take care of herself if she becomes pregnant, leaving the men’s responsibility aside [45].

### 4.3. Use of Birth Control Methods

Before pregnancy, condoms were the most commonly used contraceptive, which is in accordance with the analysis done by Allen-Leigh et al. [46] on data from ENSANUT-2012. They observed that in Mexico, the use of condoms increased from 31.8% in 2006 to 47.8% in 2012. Nevertheless, in our study, a high percentage (46.6%) of adolescent women who did not use a birth control method is demonstrated.

A reason for not using contraceptives might be that when adolescents seek birth control methods from their physician, a parent or guardian must accompany them for consent [46]. Health care providers are a common source of birth control methods for adolescents in Mexico and the rest of Latin America. However, condoms are the only method usually available in places other than medical facilities.

Another reason for not using birth control methods is the lacking knowledge regarding the different birth control options and limited access to methods other than condoms. In a sample of 7049 women from five countries of Mesoamerica, Ríos-Zertuche et al. [4] found that the use of effective contraception is low, and the knowledge about available methods is very limited among those living in poor or marginalized areas.

Another possible explanation is that health care professionals are not always updated with the most accurate information on the use of different birth control methods for adolescents. Thus, health care professionals must be better informed on the use of contraceptives by adolescents. For example, some health professionals underestimate the number of sexually transmitted diseases, as well as the frequency of pregnancies and their complications among adolescents [47]. In our study, only 5% of participants received information about birth control methods from a doctor, who usually informed the adolescent only about condoms. The rationale for this approach is that all other methods of contraception supposedly cause damage to the adolescent, which was demonstrated by Bahamondes et al. [48], who identified some deficiencies and contradictions in knowledge and attitudes based on responses from Latin American obstetricians and gynecologists who participated in the survey. In Mexico, de Castro et al. [24], conducted a study where some adolescents pretended to be clients visiting pharmacies and healthcare facilities, asking for contraceptives. The study showed that there were barriers to receiving information about contraceptives, such as administrative pretexts to avoid providing services by pharmacists and healthcare professionals. Furthermore, the participants felt judged by physicians, and stated a lack of easy and understandable information [24].

Interestingly, the group of non-users received information about birth control methods from people other than their mothers, such as a doctor or a teacher. Hence, it would be pertinent to take advantage of the communication between a mother and her daughter by including the mother in the health sector strategy for sex and reproductive education [49].

### 4.4. Postpartum Use of Birth Control Methods

A higher probability of not knowing birth control methods has been associated with initiating sexual activity before the age of 15. However, adolescents who use contraceptives before pregnancy are more likely to continue their use following childbirth. Age and pregestational use of birth control methods were also associated with postpartum use of birth control in our study.

The ENSANUT-2012 [23] reported that 52% of adolescents did not use any contraceptive after pregnancy, which continues to be true today [50]. A study on a group of U.S. women by Abraham et al. [51] showed that the use of birth control methods was similar before and after pregnancy, regardless of age or parity. Borovac-Pinheiro et al. [6] revealed similar results in a group of 196 Brazilian adolescents, of which 74% used a birth control method before pregnancy, and 76% after it (mainly hormonal treatments). The frequencies of birth control method used before and after a pregnancy in Mexico are very similar. However, in our study, we show a sharp increase due to the fact that the INPer encourages the use of contraceptives for all adolescents after childbirth [51]. At INPer, the most commonly suggested methods for adolescents after pregnancy are the intrauterine device and hormonal treatments, which differ from what adolescents used before pregnancy, where condoms were the most common [52].

Postpartum, long-acting reversible contraception remains underutilized in our universal health care system, and measures should be used to increase its use, such as improving prenatal counseling and immediate postpartum placement.

This study has some limitations. The report on the use of birth control methods was based on self-reporting, which may be subject to recall bias. Furthermore, the sample is not representative of all Mexican adolescents, the INPer brings medical attention to high-risk pregnancies that have no health care coverage, so the results cannot be extrapolated to adolescents from other regions of the country or in other countries. Another limitation could be that knowledge and beliefs regarding the use of contraceptives in adolescents from health care professionals could also affect their use [23]; however, we did not assess this issue.

One strength of this study is that the present study reveals novel and relevant information based on a group of pregnant adolescents in the urban and nearby areas of Mexico City. These results offer insights that may contribute to public policy-making aimed at increasing the use of birth control methods, and thus preventing first and later pregnancies during adolescence. Comprehensive attention for adolescents could help encourage the use of birth control methods because despite having the knowledge and sometimes the offer to use them from a healthcare professional, they still choose not to use them. This is highly important, especially in adolescents younger than 15 years, as this age group is one of the most vulnerable in Mexico and the rest of Latin America.

## 5. Conclusions

The variables that increased the risk of not using any birth control method before pregnancy were being aged under 15 years, having dropped out of school, having an educational lag, initiation of sexual life before the age of 15, and receiving information from someone other than the mother regarding birth control methods. Meanwhile, the variables that were associated with an increased risk of not using any contraceptives after childbirth were the presence of an educational lag, having a lower level of education, and that the adolescent had not used any birth control before the pregnancy.

Finally, there is no ideal method of birth control for adolescents. There are also no medical reasons for denying the use of birth control methods due to age. It is necessary to highlight the importance of adolescents having opportunities to learn about different birth control methods in order to make informed decisions about their sexual health.

## Figures and Tables

**Table 1:** Sociodemographic characteristics of the adolescents and their parents, according to users and non-users of birth control methods before pregnancy.

Characteristics	Users *n* = 318	No Users *n* = 282		*p*-Value
	Mean ± SD*		Min-max	
Age (years)	15.4 ± 1	15.2 ± 1	11–19	0.046
Age at menarche (years)	11.8 ± 1.	11.6 ± 1	8–15	0.051
Initiation of prenatal care (weeks of gestation)	16.2 ± 5	12 (8–16)	9–37	0.491
Initiation of sexual life (years)	14.5 ± 1.1	14.4 ±	10–18	0.114
Number of sexual partners **	1 (1,1)	1 (1,1)	1–5	0.694
Age of the mother of the adolescent (years) **	39 (36–46)	38(35–43)	29–58	0.722
Age of the father of the adolescent (years) **	41 (37–46)	42 (37–46)	29–68	0.709
Age of the adolescent’s mother at her first pregnancy (years) **	20 (18–23)	19 (17–23)	14–39	0.799
Age of the adolescent’s father at his first child (years) **	22 (19–25)	23 (20–26)	15–54	0.322
Age of adolescent’s partner (years) **	18 (17–20)	18 (16–19)	17–43	0.063

* Standard deviation; ** Median and interquartile range (percentile 25–percentile 75).

**Table 2:** Percentage distribution of the main topics that the adolescent were informed about at the onset of menarche (%), according to the use of birth control methods before pregnancy.

	Users *n* = 318 (53%)	Non-Users *n* = 282 (47%)	*p*-Value *	Chi-Value
Risk of pregnancy	308 (97)	265 (94)	0.134	1.187
The importance of self-care	318 (100)	265 (94)	0.003	13.791
The importance of being responsible	318 (100)	270 (96)	0.001	15.588
The possibility of infection by HIV	308 (97)	268 (95)	0.001	16.306
Birth control methods	315 (99)	26 (95)	0.029	6.058

* Pearson Chi-squared test.

**Table 3:** Percentage distribution of characteristics for adolescents who did and did not use birth control methods before and after pregnancy.

Variables		Before Pregnancy			After Pregnancy		
		Users *n* = 318	Non-Users *n* = 282	*p*-Value *	Users *n* = 382	Non-Users *n* = 213	*p*-Value *
Level of education	Low	154 (52)	214 (71)	0.001	224 (59)	143 (67)	0.041
	High	143 (48)	82 (29)		158 (41)	70 (33)	
Educational lag	No	212 (71)	155 (51)	0.001	253 (66)	113 (53)	0.002
	Yes	85 (29)	148 (49)		129 (34)	100 (47)	
Student	Yes	102 (34)	43 (14)	0.001	119 (31)	26 (12)	0.001
	No	195 (66)	260 (86)		263 (68)	187 (88)	
Source of information about birth control methods	Mother	190 (60)	147 (52)	0.034	221 (58)	117 (55)	0.490
	Other	127 (40)	135 (48)		161 (42)	96 (45)	
Occupation of the mother	Housewife	168 (58)	139 (51)	0.042	215 (56)	110 (52)	0.276
	Employee	119 (41)	134 (49)		167 (44)	103 (48)	
Socioeconomic level	Average or slightly below	214 (67)	177 (63)	0.141	265 (69)	124 (58)	0.004
	Low	104 (32)	105 (37)		117 (31)	89 (42)	
Family structure	Nuclear	207 (65)	166 (59)	0.093	242 (63)	126 (59)	0.312
	Other	111 (35)	122 (41)		140 (37)	87 (41)	
Age of initiation of sexual life	≥15 years	181 (57)	146 (52)	0.137	209 (55)	101 (47)	0.088
	<15 years	136 (43)	135 (48)		173 (45)	112 (53)	
Age	≥15 years	197 (62)	124 (44)	0.001	199 (52)	106 (50)	0.323
	<15 years	121 (38)	158 (56)		183 (48)	107 (50)	
Planned pregnancy		64 (22)	62 (19)	0.171	81 (79)	45 (21)	0.981

* Pearson Chi-squared test.

**Table 4:** Variables associated with the lack of use of birth control methods before and after pregnancy.

Variables	Before Pregnancy			After Pregnancy		
	OR	95% CI	*p*-Value	OR	95% CI	*p*-Value
<15 years old	1.950	1.284–2.973	0.002	1.084	0.701–1.679	0.716
Low level of education	1.578	1.033–2.410	0.035	1.173	0.717–1.842	0.489
Drop-out of school	2.824	1.796–4.440	<0.001	2.823	1667–4.780	<0.001
Educational lag	2.157	1.442–3.227	<0.001	1.362	0.952–2.056	0.141
Information from someone different to the mother about birth control methods	1.054	1.009–1.102	0.019	0.994	0.994–1.039	0.799
Did not use a birth control method before pregnancy				2.392	1.599–3.578	<0.001
Constant	0.019		<0.001	0.116		<0.001

## Abbreviations

ENSANUT	Encuesta Nacional de Salud y Nutrición (National of Health and Nutrition Survey)
INPer	Instituto Nacional de Perinatología (National Institute of Perinatology)
WHO	World Health Organization
Ethics approval and consent to participate	Ethical approval for the study was granted by the National Institute of Perinatology Review Board (registration number 212250-49451). Written informed consent to participate in the study, was obtained before enrollment.
